# The Treatment of Orchitis and Epididymitis

**Published:** 1881-04

**Authors:** 


The Treatment of Orchitis and Epididymitis.—Professor Wertheim recommends (*Wiener Medizinische Wochenschrift*, June 24, 1880,) in cases of moderate severity, besides rest in the horizontal position and support of the scrotum on a pillow placed between the thighs, the application of freshly spread mercurial plaster, which is to be changed every night and morning. If the swelling be very great, and the scrotum shining, and if great pain be present, ice-cold poultices are indicated. In cases of abscess, the author makes a single incision, and does not use drainage tubes, but syringes out the cavity twice a day with carbolic or other suitable lotion. When suppuration is too profuse a lotion of sulphate of copper is to be used. The emplastrum hydrargyri may be applied at the same time, an opening being made in the plaster to correspond with that of the abscess.—*London Medical Record.*

